# Supplementary material for: Pyruvate Dehydrogenase Kinase 1 inhibition mediated oxidative phosphorylation enhancement in cartilage promotes osteoarthritis progression
Source: BMC Musculoskelet Disord. 2023 Jul 20;24:597. doi: 10.1186/s12891-023-06585-6 (PMC10357736; doi:10.1186/s12891-023-06585-6)
Supplement: Supplementary file 1 — Additional file 1. [file 12891_2023_6585_MOESM1_ESM.zip › MF.Gsea.1653627316242/GOMF_PYROPHOSPHATE_HYDROLYSIS_DRIVEN_PROTON_TRANSMEMBRANE_TRANSPORTER_ACTIVITY.html]

Details for gene set GOMF\_PYROPHOSPHATE\_HYDROLYSIS\_DRIVEN\_PROTON\_TRANSMEMBRANE\_TRANSPORTER\_ACTIVITY[GSEA]

|  || Dataset | 55457\_collapsed\_to\_symbols.cls.cls #OA\_versus\_normal.cls.cls #OA\_versus\_normal\_repos |
| Phenotype | cls.cls#OA\_versus\_normal\_repos |
| Upregulated in class | OA |
| GeneSet | GOMF\_PYROPHOSPHATE\_HYDROLYSIS\_DRIVEN\_PROTON\_TRANSMEMBRANE\_TRANSPORTER\_ACTIVITY |
| Enrichment Score (ES) | 0.65085936 |
| Normalized Enrichment Score (NES) | 1.97971 |
| Nominal p-value | 0.0022624435 |
| FDR q-value | 0.01010993 |
| FWER p-Value | 0.128 |
Table: GSEA Results Summary

  

Fig 1: Enrichment plot: GOMF\_PYROPHOSPHATE\_HYDROLYSIS\_DRIVEN\_PROTON\_TRANSMEMBRANE\_TRANSPORTER\_ACTIVITY      
 Profile of the Running ES Score & Positions of GeneSet Members on the Rank Ordered List

  

| SYMBOL | TITLE | RANK IN GENE LIST | RANK METRIC SCORE | RUNNING ES | CORE ENRICHMENT || 1 | ATP6V1C1 | ATPase H+ transporting V1 subunit C1 [Source:HGNC Symbol;Acc:HGNC:856] | 17 | 1.125 | 0.1502 | Yes |
| 2 | ATP6V0D1 | ATPase H+ transporting V0 subunit d1 [Source:HGNC Symbol;Acc:HGNC:13724] | 135 | 0.872 | 0.2586 | Yes |
| 3 | ATP6V1E1 | ATPase H+ transporting V1 subunit E1 [Source:HGNC Symbol;Acc:HGNC:857] | 287 | 0.758 | 0.3491 | Yes |
| 4 | ATP6V1G1 | ATPase H+ transporting V1 subunit G1 [Source:HGNC Symbol;Acc:HGNC:864] | 515 | 0.655 | 0.4197 | Yes |
| 5 | ATP6V0E2 | ATPase H+ transporting V0 subunit e2 [Source:HGNC Symbol;Acc:HGNC:21723] | 532 | 0.647 | 0.5056 | Yes |
| 6 | ATP6V1D | ATPase H+ transporting V1 subunit D [Source:HGNC Symbol;Acc:HGNC:13527] | 750 | 0.581 | 0.5670 | Yes |
| 7 | ATP6V0E1 | ATPase H+ transporting V0 subunit e1 [Source:HGNC Symbol;Acc:HGNC:863] | 1207 | 0.474 | 0.5956 | Yes |
| 8 | ATP6V1H | ATPase H+ transporting V1 subunit H [Source:HGNC Symbol;Acc:HGNC:18303] | 1304 | 0.457 | 0.6497 | Yes |
| 9 | ATP12A | ATPase H+/K+ transporting non-gastric alpha2 subunit [Source:HGNC Symbol;Acc:HGNC:13816] | 2330 | 0.323 | 0.6137 | Yes |
| 10 | ATP6V0C | ATPase H+ transporting V0 subunit c [Source:HGNC Symbol;Acc:HGNC:855] | 2790 | 0.271 | 0.6145 | Yes |
| 11 | ATP5F1B | ATP synthase F1 subunit beta [Source:HGNC Symbol;Acc:HGNC:830] | 3024 | 0.247 | 0.6298 | Yes |
| 12 | ATP4B | ATPase H+/K+ transporting subunit beta [Source:HGNC Symbol;Acc:HGNC:820] | 3160 | 0.234 | 0.6509 | Yes |
| 13 | ATP6V1F | ATPase H+ transporting V1 subunit F [Source:HGNC Symbol;Acc:HGNC:16832] | 3833 | 0.173 | 0.6220 | No |
| 14 | ATP6V1B2 | ATPase H+ transporting V1 subunit B2 [Source:HGNC Symbol;Acc:HGNC:854] | 7074 | -0.086 | 0.3821 | No |
| 15 | ATP6V1A | ATPase H+ transporting V1 subunit A [Source:HGNC Symbol;Acc:HGNC:851] | 8013 | -0.156 | 0.3303 | No |
| 16 | ATP4A | ATPase H+/K+ transporting subunit alpha [Source:HGNC Symbol;Acc:HGNC:819] | 10417 | -0.363 | 0.1927 | No |
Table: GSEA details [plain text format]

  

Fig 2: GOMF\_PYROPHOSPHATE\_HYDROLYSIS\_DRIVEN\_PROTON\_TRANSMEMBRANE\_TRANSPORTER\_ACTIVITY      
 Blue-Pink O' Gram in the Space of the Analyzed GeneSet

  

Fig 3: GOMF\_PYROPHOSPHATE\_HYDROLYSIS\_DRIVEN\_PROTON\_TRANSMEMBRANE\_TRANSPORTER\_ACTIVITY: Random ES distribution      
 Gene set null distribution of ES for **GOMF\_PYROPHOSPHATE\_HYDROLYSIS\_DRIVEN\_PROTON\_TRANSMEMBRANE\_TRANSPORTER\_ACTIVITY**
